# Perception of School Committee Members (SCMs) on Factors Contributing to Overweight and Obesity Among High School Students in Kiribati: A Qualitative Study

**DOI:** 10.3389/fpubh.2022.754111

**Published:** 2022-04-11

**Authors:** Tanebu J. Tong, Masoud Mohammadnezhad, Nasser Salem Alqahtani, Mosese Salusalu

**Affiliations:** ^1^Department of Public Health, Ministry of Health and Medical Services, South Tarawa, Kiribati; ^2^School of Public Health and Primary Care, Fiji National University, Suva, Fiji; ^3^Department of Clinical Nutrition, Northern Border University, Arar, Saudi Arabia

**Keywords:** high school, adolescents, overweight and obesity, determinants, School Committee Members, Kiribati

## Abstract

**Background:**

Schools are vital settings for overweight and obesity prevention among children and adolescents. School Committee Members (SCMs) are crucial assets to engaging students with overweight and obesity prevention programs. This study aimed to determine factors contributing to overweight and obesity among high school students in Kiribati through the perception of SCMs.

**Methods:**

This prospective qualitative study was conducted in four randomly selected senior high schools in South Tarawa, Kiribati, from August to November 2020. With a purposive selection of 20 SCMs employed at the four high schools, both male and female participants consented to participate in the study. A semi-structured open-ended questionnaire was used for data collection using focus group discussions (FGDs). Data were transcribed and analyzed using the thematic analysis method.

**Results:**

Twenty participants were involved in FGDs with equal number of SCMs (n = 5) who attended FGDs for each school and 45% of them were female participants. Six themes were identified, namely, knowledge, behaviors, perceived status toward overweight and obesity, perceived action benefits, perceived barriers to practices, and proposed strategies to overweight and obesity prevention. These themes reveal that SCMs have a broad understanding and skill set for overweight and obesity causes and effects. However, the aptitude alone is not enough to prevent the occurrence, and thus, proposed feasible plans were voiced for responsible stakeholders to include in policy developments for overweight and obesity prevention.

**Conclusion:**

This study recognized that the knowledge-behavior gap is the main reason behind the failure in preventative strategic approaches among adolescents. As role models to students, SCMs and their schools should team up in implementing the public health policies and building mutual awareness and understanding with students and other specialist stakeholders for a more momentous and viable impact.

## Background

Overweight and obesity has become a major public health issue in all societies among children and adolescents (1). Further delay in feasible preventative strategies will enable body weight to increase at an individual level with advanced contribution to the nation's detrimental health consequences and national economic instability (2). The global prevalence of overweight and obesity is increasing. According to Yatsuya et al., overweight and obesity exists in more than half the population in America (61.1%), Europe (54.8%), and Eastern Mediterranean (46.0%), while it is 26.9% in Africa and 13.7% in South-East Asia (3). Determinants of overweight and obesity among adolescents range from socioeconomic background to unhealthy behavioral lifestyle choices such as skipping breakfast, high screen-view time, high energy food consumption and low fruit and vegetable intake, and physical inactivity (4–6). Although preventable, the detrimental health consequences of overweight and obesity as known worldwide vary. The psychosocial distress of weight stigma and mood disorder, to name a few, together with health impacts of non-communicable diseases (NCDs), premature deaths, and disabilities pose risk to the current health of society (7–9). Since the basis of occurrence is multifactorial, the prevention requires comprehensive strategies and multiple stakeholders to control this widespread public health concern (10, 11).

Overweight and obesity among adolescents in the Pacific region is reported to increase. Reports from the Global Burden of Disease (GBD) study 2013 stated that body mass index (BMI) of greater than the 85th percentile on BMI for age chart showed levels in Fiji to be 12.8% among boys and 24.9% among girls. The Solomon Islands presented statistics of 28.3% among boys and 49.2% among girls, while Tonga relates to 52.6 and 34.5% among girls and boys, respectively (12). Vanuatu also stated in the GBD study that 14.5% of young boys and 23.2% of young girls were also among the high BMI levels (13). Moreover, Samoa reported 42.2% among boys and 50.0% among girls (14). Overweight and obesity is the major issue in Kiribati where most of its population is facing the consequences leading to the high risk of decreased life expectancy and poor management within the health sector. Reports from Global statistics (2015) mentioned that the highest prevalence of overweight and obesity among Pacific Islands Countries (PICs) is found in Kiribati with 47.7 and 66.1% among boys and girls, respectively (15).

The obesogenic environment due to modernization of diet and increase in energy-dense diet coupled with a reduction in daily energy expenditure due to sedentary lifestyles is now more common among high school students globally (16). This phenomenon is globally reported in western cultures and is currently expanding into developing countries in the Pasifika small countries (17–19).

Multiple studies have stated that a significant aspect influencing the effectiveness of overweight and obesity prevention is the role of School Committee Members (SCMs), which comprise principals, deputy principals, teachers, and parents, who implement knowledge through their expertise and being immediate role models to students (20–22). The perspectives of SCMs are very important to explore and address in relation to overweight and obesity since SCMs play a significant responsibility in providing guidance, becoming effective leaders, and being positive role models to high school students (23). A study by Howard-drake and Halliday (2015) states that headteachers are crucial partners in overweight and obesity prevention (24).

**Figure 1:**
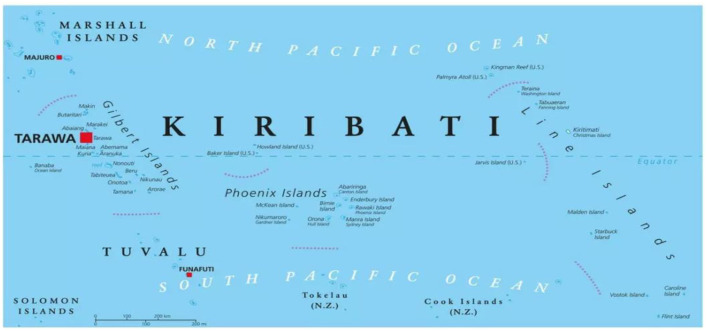
Map of Kiribati Islands (25).

This study was conducted in the Gilbert Islands, currently known as the Republic of Kiribati, an independent country since 1979, situated in the central Pacific Ocean, with its largest capital island of South Tarawa (Figure 1). It is positioned along the equator of where the international dateline passes through (26). It is a small island nation that consists of 33 atolls that are scattered across ~3.45 million square kilometers of the ocean. The population according to the 2018 census is roughly around 115,850 of which more than half the population residing on the capital island of South Tarawa (27), that is, about 15 km long and between 300 and 500 m wide, with the highest point being 3 m above sea level. It is a resource-limited country that carries a burden of both communicable and non-communicable diseases (28). Life expectancy is around 69 years for both genders with 73 years in women and 67 years in men (29).

Kiribati is lacking in literature based on the perspectives of principals, teachers, and school administrators in terms of knowledge, understanding, and what influences their decision-making processes with overweight and obesity among high school students. The aim of this qualitative study was to explore factors contributing to overweight and obesity among high school students in Kiribati through the perspectives of SCMs to reduce the gap in the literature and understand perceptions on knowledge-behavior gap recognized by other studies as the main reason behind the failure in lifestyle changes among adolescents and, in addition, to explore variables needing school setting regulation and healthy food policy coupled with knowledge and behavior of school children at school level.

## Materials and Methods

### Study Design and Setting

This study used a prospective qualitative approach where SCMs in groups of five attended focus group discussions (FGDs) assigned to one of the four randomly selected high schools with respect to their workplace. The FGDs were conducted between August and November 2020 in a resource-limited Pacific Island that lies roughly between Hawaii and Australia and ranks among the highest in NCDs related to overweight and obesity. The four selected senior high schools included Saint Patrick College (StPC) at the far west, Moroni High School (MHS) and William Goward Memorial College (WGMC) toward the central division, and King George V & Ellaine Bernacchi School (KGV & EBS) toward the east.

### Study Sample

This study included all SCMs from senior high schools in South Tarawa. The inclusion criteria for the SCMs included the teachers, school managers/principals, together with religious and government administrators working in participating senior high schools. The exclusion criteria were those working in non-selected high schools, those who have participated in the pilot study FGD, and those unwilling to participate in FGDs. Those who met the study criteria were invited to participate in FGDs.

A purposive sample approach was used to select 4 to 5 SCMs to participate from each randomly selected school. SCMs were also selected for maximum variation, availability, and data richness where a total of 20 SCMs were grouped according to the schools each represent and participated in respective FGDs that enabled participants to openly talk with other group members and research assistant.

### Data Collection Tool

A semi-structured questionnaire with open-ended questions was developed using the aims and objective of this qualitative design with a clear concept and aim to grasp the first-hand experience of SCMs. The questionnaires consist of two sections: the first part is a demographic summary of the participant with issues relating to gender, residing village, job, and religion, while the second part consists of six open-ended questions on factors contributing to overweight and obesity among high school students in Kiribati. The questionnaire was available in English but was also translated to Kiribati using bilingual translators. This translated version was translated back to English using a different translator to guarantee contents of both English and Kiribati versions were consistent with each other.

### Study Procedure

After all ethical approvals and endorsements were sorted and cleared, a trained research assistant was recruited and consented for confidentiality issues to assist in data collection. All SCMs from the four randomly selected high schools were informed about the research and were all invited to participate. Information sheets were given to ensure understanding, confidentiality, and option to withdraw anytime during the duration of research. A consent form was also given to SCMs to sign, and upon receipt of all consent forms, the date, time, and venue of FGD were arranged with the principals and deputy principals of high schools. All FGDs took ~1 h and 30 min, and notes were handwritten and audiotaped.

### Data Management and Analysis

The research assistant had audio recorded and manually handwritten discussions made during FGD and sent to the primary researcher for transcription. The research assistant verified all transcriptions for accuracy. Upon confirmation of transcription, data were entered into Microsoft Excel where important keywords and phrases were labeled, grouped, and coded with FGD number and high school represented. Thematic analysis using Microsoft Excel, as described by Bree and Gallagher, was used (30) where the data collected were coded and cleaned and a thematic framework was developed. First, a two-column table is developed where the first column has the original transcript and the second column consists of codes that are further cleaned to make each code consistent with the original transcript. Second, from the consistent codes, a thematic framework is developed with respect to the conceptual framework and research questions.

### Study Rigor

A trustworthy research is considered an appropriate benchmark for evaluating a qualitative study. As Guba and Lincoln proposed, a research should satisfy credibility, transferability, dependability, and conformability (31). For the credibility of data collected, three teleconference calls were held for training purposes. Also, pilot interviews among the research team were conducted to ensure time management, feasibility, and comprehensibility. This study has recruited a qualified research assistant with background experience of investigative knowledge, skillful with large datasets, and with multidisciplinary tasks of seniority, nursing, and public health graduate.

### Ethical Considerations

Prior proceeding with research, ethical approvals were obtained from the College Health Research Ethics Committee (CHREC) at the Fiji National University (FNU), from the Ministry of Education (MoE) and the Ministry of Health and Medical Services (MoHMS) Research Ethics Committee in Kiribati, and Principals and Deputy Principals of the participating high schools. The research assistant and all participating SCMs were informed of the study purpose, consented for participation, and ensured confidentiality, protection, and security of identities. Informed consents were obtained for SCMs before collecting data.

## Results

### Demographics and General Characteristics of SCM

Twenty participants were involved in FGDs with equal number of SCMs (*n* = 5) who attended FGDs for each school, of which 45% were female participants and 55% were male participants. All SCMs lived across the capital island mostly in the populated villages of Bikenibeu 30%, Teaoraereke 20%, and Betio 20%. The majority of participants were 50% teachers by profession, and the dominant religion was 50% (Table 1).

**Table 1:** General characteristics of SCMs (*N* = 20).

**Variables**		**Frequency**	**Percentage**
High school	KGV & EBS	5	25
	MHS	5	25
	WGMC	5	25
	*StPC*	5	25
Gender	Female	9	45
	Male	11	55
Residential village	Temaiku	1	5
	Bikenibeu	6	30
	Eita/Taborio	2	10
	Teaoraereke	4	20
	Bairiki	3	15
	Betio	4	20
Position/job	Principal/Deputy	4	20
	Teacher	10	50
	Church representative	3	15
	Community representative	3	15
Religion	Roman Catholic	10	50
	Latter Days Saints	5	25
	Kiribati United Church	5	25

*KGV & EBS: King George V & Ellaine Bernacchi School; MHS: Moroni High School; WGMC: William Goward Memorial College; StPC: Saint Patrick College*.

### Themes and Subthemes

Focus group discussions were done in the four selected schools with five members from each school. Six main themes were identified from FGDs, namely, knowledge, behaviors, perceived status toward overweight and obesity, perceived action benefits, perceived barriers to practices, and proposed strategies to overweight and obesity prevention. The citation of responses will be group number and school, for example, FGD 1, KGV, and EBS (Table 2).

**Table 2:** Themes extracted from FGDs.

**Themes**	**Subthemes**
Knowledge toward overweight and obesity	Understanding the cause of overweight and obesity
	Perceived impact of overweight and obesity
	Proposed strategy to prevention
Behaviors toward overweight and obesity	Stigma toward people with overweight and obese
	Overeating
	Not physically active
	Ignorant
	Weight loss
Perceived status of overweight and obesity	Modifiable risk factor
	Disease
	Detrimental effect
Perceived action benefits	Reduce stress and anxiety
	Improve health
	Improve self-confidence
Perceived barriers to practices	Stigmatization
	Low motivation
	Lack of understanding
Proposed strategies to overweight and obesity prevention	Home improvement
	School support
	National sustenance

### Theme 1: Knowledge Toward Overweight and Obesity

There are three subthemes identified under the theme of knowledge. These subthemes are understanding the cause, perceived impact, and prosed strategy to preventing overweight and obesity.

#### Understanding the Cause of Overweight and Obesity

Understanding the experiences of people reflects very much on how each cope with daily challenges. When social committee members were asked about what they know about overweight and obesity, three groups mentioned straight after the question that it is all about overeating and less exercise. However, one group mentioned knowledge and understanding not matching up to actions and practices:

*I think that for us adults, it is hard to spend time working out or exercising… It is becoming a bad habit that is affecting everyone and everything. We know and understand the cause of overweight and obesity, but our actions and practices seem dull*. (FGD 2, MHS)

Furthermore, one of the groups said that overweight and obesity is having a high BMI, but the energy and strength to decrease the BMI to healthy weight are complicated:

*The knowledge on overweight and obesity does exist in everyone but the will power to return to healthy weight is really difficult to practice. I try to find time to exercise but it seems difficult with all the work I must do. However, if I have free time, it is used for something unproductive*. (FGD 1, KGV & EBS)

Another contributing factor to overweight and obesity is the amount of food intake. It is a norm to eat as much as you can without proper food servings or even the presence of vegetables in a meal. One participant mentioned that reminiscing back on the type of diet he had since childhood, hardly there was neither any vegetables nor any fruits:

*I think there is essential knowledge missing in the whole overweight and obesity picture. Although part of the knowledge is there, the vitals are missing. We must start practicing now, the proper way of feeding our children so when they grow up, society will change that instead of eating till you drop, you eat just enough but well balanced*. (FGD 3, WGMC)

One FGD discussed about the importance of defining needs and wants and differentiated among the two for a better understanding of financial crisis among families:

*Another aspect that is missing is financial expenditure of a family. Most families claim that they are always in dilemma as to what needs to be bought. However, the majority end up spending more on wants than needs. We need to revisit the needs and wants and practice more on how to deal with each*. (FGD 4, StPC)

Another missing aspect mentioned by one group is the utilization of the free hospital services offered by the government. Hardly anyone goes for routine checkups. The only time one would visit the hospital is when they feel the need to. No one attends the clinic on regular basis:

*I don't see the need to attend clinics just for medical checkup. I will only go to the hospital when I feel the need to. Even if we go to the hospital for an occasionally checkup, the hospital staffs are not at all welcoming or helpful especially when they see a healthy person requesting medical checkup. I believe they are already stressed with work and do not wish to waste their time on non-sickly-looking people*. (FGD 1, KGV & EBS)

The hospital protocols are always superseded by the public influence and demand. As one FGD conversed, people are only seen at the hospital when severely sick or distressed. Moreover, if non-sick people require a medical checkup, the proper procedures are never followed and instead request to see the doctors for immediate assistance:

*If we need medical checkup, we tend to request to see doctors only. There is also a proper medical channel that we the public is not aware of and that is, to see the receptionist first, then nurses, then medical assistants, and lastly doctors when there is urgent need. Most times we are demanding and rude*. (FGD 4, StPC)

#### Perceived Impact of Overweight and Obesity

When asked about the effects of overweight and obesity in life, all four groups remained quiet. One group repeated the question to the facilitator:

*I don't really know the effect of overweight and obesity in life. Maybe continue to diabetes? Not sure*. (FGD 1, KGV & EBS)

Some members of the FGD are not aware of overweight and obesity definition, cause, or effect:

*Can we die because of overweight and obesity? I don't really know, I am only asking*. (FGD 4, StPC)

Public awareness on health issues, as well as integrating health science into the high school curriculum, was recommended by one FGD:

*Maybe we can request health talks on overweight and obesity to schools for students and teachers to grasp a full context about. Health science should be another elective course for students to take but more in-depth for high school level*. (FGD 2, MHS)

Another FGD was quiet and incompetent around the issue discussed:

*I don't want to say anything as I am afraid it will be wrong. Our group does not want to give false beliefs*. (FGD 3, WGMC)

#### Proposed Strategies for Prevention

The preventative measures of overweight and obesity as mentioned by the FGD participants were targeted at every level. One group aimed at families, communities, and schools, while two groups mentioned the government and stakeholders. The last group, however, focused on the individual self:

*I believe members of the communities should work together to combat overweight and obesity. Motivate one another through physical activities and balanced diets. Finding a strategy most fitting to each community and implementing it slowly can be a start of the battle. The only problem is, what may happen when the battle is long, and members of the community begins to lose hope?* (FGD 3, WGMC)

One FGD emphasized the need to decrease marketing costs on healthy foods and to build facilities to cater the need of exercising indoors:

*Prevention is better than cure or so they say in health visits to the communities. The government and other stakeholders should invest in gymnasiums or even so, subsidize the cost of nutritional foods on the market*. (FGD 1, KGV & EBS)

Another FGD requested for plantations to create jobs for people and to have locally produced vegetables for sale on the island:

*Why not ask the government to invest in planting vegetables to sell for cheap. In this way, more jobs can be made available and furthermore, nutritional diets can be sold for cheap if locally planted*. (FGD 2, MHS)

Self-change and motivation are another issue raised by one FGD:

*If we can change our behaviors towards overweight and obesity, the result will also change. Motivation to the core is essential in this matter*. (FGD 4, StPC)

Despite all that has been discussed and shared in groups, another responded that overweight and obesity is a norm in Kiribati. The majority believed that big is beautiful in the Pacific and that the need to maintain good hygiene and health remains in the hands of the individual:

*If you feel happy and healthy in this life you are good and beautiful. I do not want to believe what others tell me. I will experience life to the max*. (FGD 3, WGMC)

Due to cultural norms in the Pacific practice, one FGD advocated for strategies to teach the younger generation about the importance of physical activity and healthy diet:

*This is the first time I hear big is overweight or obesity. Thinking about it, it scares me of the result. Trying to slim down will require a lot of work and effort*. (FGD 1, KGV & EBS)

### Theme 2: Behaviors Toward Overweight and Obesity

Five subthemes, namely, stigma, overeating, not physically active, ignorant, and weight loss, have been recognized under this theme.

#### Stigma

Behaviors of adults in the family play an important role in young people's lives. However, adolescents can decide for themselves but still require the guidance of parents and guardians. All groups realized that their behavior is not in favor of overweight and obesity prevention:

*Despite the knowledge already attained, our behaviors do not change. It is really difficult to change these behaviors right away; in fact, it needs time to change*. (FGD 2, MHS)

One group mentioned that it all depends on an individual's behavior for change to occur. Everyone must change, then the society's perspective will also change:

*I can change my behaviors and actions towards a concerning issue but changing others will be a challenge. Sometimes, the strive to change fails and everyone is back to square one*. (FGD 1, KGV & EBS)

#### Overeating

One group expressed the concept behind the phrase “you are what you eat” and the need to change:

*Nothing is complicated in this world. We are the cause of what we need to change*. (FGD 1, KGV & EBS)

The concept of overeating is interpreted in different ways and has affected the society negatively:

*This group wants to know what is meant by overeating because to some, it is when you combine breakfast, lunch, and dinner in one meal. Others say that it is just a catchup meal for all those missed during the day*. (FGD 2, MHS)

#### Not Physically Active

While discussing exercise and other physical activities, two FGDs really emphasized that no physical activity has a major impact on overweight and obesity production:

*If we eat and don‘t do exercise, which is common on South Tarawa, the outcome is big size or increase in weight. There is no other explanation to the situation*. (FGD 4, StPC)

The third FGD member emphasized on stored energy:

*With the current unhealthy eating habits, the weight just adds on*. (FGD 3, WGMC)

#### Ignorant

Another group stated that being ignorant is a problem just like any other issues. If one does not worry about the other but focus on own family, the attempt to behavioral change may or may not begin:

*I think we must not worry about others. Just focus and maintain good health in our immediate families and hopefully the change to society will occur over time*. (FGD 4, StPC)

One group understood that change can be done when beneficial for family:

*We don't want to change for others, we need to change for the benefit of family and myself*. (FGD 3, WGMC)

#### Weight Loss

When asked about the whole perspective on overweight and obesity, all four groups mentioned that it is truly a challenge caused by people themselves. Although behavioral change is the solution, the implementation seems so complicated:

*I don't know why but weight loss activities are time consuming, difficult to maintain, and results hardly show instantly*. (FGD 2, MHS)

Another group requested immediate action to change the panorama of overweight and obesity on mankind:

*Overweight and obesity is simple yet complex to many. The outcome however remains dreadful. We need to act now if we want to change the future*. (FGD 4, StPC)

### Theme 3: Perceived Status of Overweight and Obesity

The subthemes recognized under this theme are modifiable risk factors, the disease, and detrimental outcome.

#### Modifiable Risk Factors

The largest contributing factor to overweight and obesity is the modifiable risk factor, and several FGDs highlighted this aspect:

*Behavioral lifestyles such as exercise, sleep, and unhealthy food habits to name a few are modifiable but requires determination*. (FGD 1, KGV & EBS)

In their discussion, one FGD stated as follows:

*Consuming sweetened food and beverages are common among children and adolescents. These junk foods can be eliminated from school canteens but would require public awareness*. (FGD 2, MHS)

#### The Disease

The most important aspect of overweight and obesity is the difficulty to prevent despite being modifiable. This would require a multifactorial approach:

*Although modifiable risk factors are hard to prevent, approaching the disease in multiple areas is a reasonable approach*. (FGD 3, StPC)

Another FGD member recognized that despite several efforts and attempts to preventative measures, there is still no definite strategy available:

*Despite public health campaigns on preventative measures to overweight and obesity, there is no clear-cut method feasible in homes, schools, nor communities*. (FGD 1, KGV & EBS)

#### Detrimental Outcome

The status of overweight and obesity that was discussed has been threatening to some FGD members. There is no turning back once the end point is reached. The harm and disheartening outcome are a rising concern:

*I think overweight and obesity is a risk that contributes to premature deaths and disabilities of society. It is really complicated when thinking about it since the upbringing and background from the very beginning emphasized and practiced that being big is beautiful. It is about time to change this perspective*. (FGD 2, MHS)

One member from an FGD relayed the risk their family has been carrying and poses a risk on the confused definition of being big:

*I come from a family lineage of big family members and luckily no one is diabetic or hypertensive. This does not mean we are healthy, but it means that we are at great risk to diseases*. (FGD 3, WGMC)

One member from another FGD expressed sympathy to families and friends who have lost their lives due to the complications of overweight and obesity:

*I am afraid to say that overweight and obesity is a risk factor of multiple diseases. I have seen many of my friends passed this lifetime so young*. (FGD 4, StPC)

### Theme 4: Perceived Action Benefits

The perceived action benefits encompass three subthemes, namely, reduce stress and anxiety, improve health, and improve self-confidence.

#### Stress and Anxiety Reduction

Actions and practices to overweight and obesity are effective and reduce stress and anxiety as many participants in FGDs have mentioned. However, knowledge alone is not enough; rather, both actions and practices must exist to complete the cycle:

*If we know that we need to eat on daily basis but do not practice, we will surely expire over time. There is no point in knowing and not doing. It is surely stress-free to know and to practice*. (FGD 1, KGV & EBS)

#### Improve Health

From the FGDs, all groups believed in the need to implement knowledge and understanding. However, society practices what they see only in reality. The change to everything that involves overweight and obesity takes time for the outcome to show, and thus, individuals loose interest even before any change can occur:

*I have attempted to exercise more, however when I see the change in body, I slack in workout. Surprisingly over 48 hrs I start to gain weight again. Takes time to get rid but an instant to gain. This is what makes me give up*. (FGD 3, WGMC)

It is tough to maintain physical fitness as the motivation always slacks. One FGD believed in continuous physical activity to maintain endurance and become part of a normal routine:

*Once you start your workout, the idea here is not to stop. Over time your body will eventually get use to the exercise, and it will no longer become a burden*. (FGD 2, MHS)

#### Improve Self-Confidence

Being fit and healthy improves self-esteem and confidence as mentioned by all FGD members:

*Knowing that we are within healthy weight makes us feel happy and confident. We can sense some of our FGD members feel downhearted knowing the causes and effects of overweight and obesity on society*. (FGD 3, SHC)

Another FGD member stated that:

*Being a teacher makes me happy because I can guide students to fitness. I just need to build confidence by keeping fit myself*. (FGD 4, StPC)

### Theme 5: Perceived Barriers to Practices

The subthemes identified under perceived barriers to practices are stigmatization, low motivation, and lack of understanding. These subthemes were further elaborated by the voices of SCMs.

#### Stigmatization

Barriers to practices are many but self-motivation tops the list. The second barrier on the list is the lack of public awareness. The third barrier is the need for support in terms of motivation and public awareness. The will power to continue exercise is also vital in these issues; otherwise, there is no point in the workout:

*I need to exercise but I can only do it now with group of friends. Most times I feel lazy and want to sleep more hours than needed*. (FGD 3, WGMC)

One FGD elaborated on imitation but encourages everyone to ignore and maintain good health:

*Mockery is common in Kiribati and exercising will be perceived differently. It is never too late to attempt and maintain exercise*. (FGD 1, KGV & EBS)

#### Low Motivation

It is demotivating to initiate physical fitness in an environment that is not used to physical activity:

*I am eager to become fit but first I need to build confidence and be motivated to start any feasible programs available*. (FGD 1, KGV & EBS)

Another group preferred being in groups as being alone only gives them low motivation:

*I require a group of friends to join any weight loss program in order for me to become inspired and motivated to join*. (FGD 3, WGMC)

#### Lack of Understanding

Another FGD discussed the limitation of knowledge as a barrier and highlighted the need for communities to be enlightened on the issue:

*Lack of understanding is another barrier to practices. Many community members do not know why they need to exercise. Life is complicated in so many ways…would request those responsible to ensure public awareness on the issue*. (FGD 2, MHS)

Knowledge and understanding are the key to success in all issues and concerns. The public should be made aware of the importance of physical exercise and healthy diet as one FGD explained:

*I would request the health team to visit homes, communities, and schools for public awareness. This may decrease ridicule comments and break the barrier to beneficial practices*. (FGD 4, StPC)

Public awareness includes all communities and institutions within. One FGD requests extra practical curriculums to be integrated into high school courses for untiring consciousness:

*If there were a possibility, I would request MoE to add Health science and physical education as an elective course in high school*. (FGD 1, KGV & EBS)

### Theme 6: Proposed Strategies for Overweight and Obesity Prevention

Proposed strategies for overweight and obesity prevention highlighted three main subthemes, namely, home improvement, school support, and national sustenance.

#### Home Improvement

Overweight and obesity prevention is complex. Governments across the globe are finding difficulty combating this modifiable risk factor of many diseases. Discussions made by SCMs were able to highlight some important factors deemed feasible in resource-limited areas. One group stated as follows:

*To even think about the word prevention, it should start from a home. I know that the support from home is extraordinarily strong because the first 5 years of childhood life is vital in paving the future lifestyle of a child. Therefore, I would suggest strategies most fitting to a home environment to make the first move of change*. (FGD 3, WGMC)

Food preparation with healthy foods is another simple activity that can be implemented at home. According to a statement made by an SCM:

*To even attempt cooking healthy food is the first step in prevention. It is critical to find balanced edible green leaves that are cheap and readily available to add into meals of our family. It doesn't have to be anything fancy. All we need to keep in mind is the health and happiness*. (FGD 1, KGV & EBS)

At times, family discussions can be difficult due to culture, but it is the most influential task children learn from:

*I remembered speaking to my daughter about overweight issues and she responded that if I will support her in diet and sport, then she too would drag me to do the same. Life and experiences are fun when shared together*. (FGD 4, StPC)

Looking around to see what others are doing is also amusing that sometimes activities are automatically in place:

*My family does not have a home garden, but our neighbors do. My son admires their garden and the next few weeks, we have a garden at home. We are now looking forward to eating fresh papaya, tomatoes, and green cabbage when harvest time arrives*. (FGD 2, MHS)

A balanced diet in a home builds on a taste that most of the time seems normal when everything else is tasteless. One FGD discussed the past experience in times of food insecurity:

*We are lucky we drink water more than sweetened juice. Our meals are simple with just the right portion size. One time there was no sugar on the market and people were rushing from one store to another. I see the difference between their struggle and my family*. (FGD 4, StPC)

#### School Support

School support in issues concerning students is vital. The need for curriculums and policy development for schools is recommended for SCMs to implement. One FGD discussed and mentioned as follows:

*There is no curriculum that favors overweight and obesity prevention. Physical education, Nutritional Health, and Agriculture is not among the topics studied. It is different from schools around the region. I am wondering, what is the current prevalence of obesity and overweight among our students and staff. Would be interesting to know*. (FGD 1, KGV & EBS)

Some teachers are not bothered by weight gain. However, their interest in gardening has made the school bloom with flowers. School garden in terms of fresh fruits and vegetables can be very influential:

*I noticed that many teachers in school like planting flowers as a hobby. I wonder if they can share their talent but in terms of fruits and vegetables so this school can be an example to other schools blooming with healthy foods*. (FGD 2, MHS)

#### National Sustenance

Kiribati is a resource-limited country that is undergoing the effects of climate change. However, considering overweight and obesity, it is the voice of the government that can influence the people in living a healthy lifestyle:

*Public awareness by respective stakeholders is critical in combating the impact of overweight and obesity. Although budget is limited, the there are many cost-effective methods that can be used such as social media, newspapers, and youth drama groups*. (FGD 3, WGMC)

There are many strategies feasible in Kiribati as voiced by students and SCMs. However, the approaches are all reliant on teamwork and time management:

*The reason we cannot accommodate extracurricular activities in school curriculums is because of time spent in fundamental courses. I think it is true that school is so relaxing to several students and especially to teachers. I would opt for extra classes if it reasons favor good health to the young population*. (FGD 1, KGV & EBS)

## Discussion

The main important findings of SCMs in this study were the behavioral lifestyles common among adolescents that persisted through adulthood (32). Moreover, the perceptions of SCMs were clustered into six themes, namely, knowledge, behaviors, perceived overweight and obesity status, perceived benefits of action, perceived barriers to practices, and proposed strategies for overweight and obesity prevention.

### Knowledge Toward Overweight and Obesity

Understanding the cause of overweight and obesity, perceived impact, and proposed strategies to prevention were the main discussion among SCMs of the four FGDs. The participants of one FGD mentioned that lack of time management, especially relaxing on screen media, was the main cause of overweight and obesity among adolescents. Similar research by Robinson et al. and Hurby et al. highlighted that the number one outcome of screen modes exposure is overweight and obesity (33, 34).

Moreover, the FGD members perceived premature deaths and disabilities as an undesirable impact of overweight and obesity in society. However, the negative influence of overweight and obesity among adolescents as mentioned by all FGD is weight discrimination, social isolation, and body dissatisfaction. This brings into line the results by Spahlholz from a meta-analysis on discrimination and overweight (35), with more literature on isolating from society (36, 37) and body distortion (38, 39). The disparity is the perception of the impact of overweight and obesity. This shows that Kiribati is worried about premature deaths and disabilities as seen often due to ignorance of the public in seeking medical attention early.

Another point to discuss is the essentials of missing link to overweight and obesity is the upbringing of a child at home from birth. Abidin explained that children could combat overweight and obesity through the introduction of a nutritious diet early in life (40).

### Behaviors Toward Overweight and Obesity

Attitude and behavior to many health issues are usually unfavorable compared with the knowledge revealed. One concerning behavior of students voiced by one FGD in this study was the definition of needs and wants among adolescents. The majority of students will not buy fruits or drink water due to cost and taste, respectively. However, the budget for junk food and other unnecessary substances is readily available without hesitancy. The behaviors among students and the socioeconomic background of parents as highlighted in this study are parallel to the literature reviewed. Borraccino et al. and Babajafari et al. stated the increased consumption of junk food seen among students with low socioeconomic status (41) These two studies highlight and support overweight as more prevalent in the low-income state.

Moreover, another FGD stated that students in Kiribati have a short span of willingness. Therefore, if the weight-loss strategy were to show effect in a matter of hours, then the power to exercise will be in full capacity. Unfortunately, weight loss requires days and weeks to be effective but the span of motivation and determination in the majority of students is not prolonged but in fact unpredictable. Findings of this study are related with the literature reviewed. Previous studies have repeatedly shown that weight loss is motivated by biological factors, psychological influences, as seen in this study, and social aspects (42, 43). However, Wadden and Stunkard stated the need to improve motivation to lose weight. This determines that having less motivation doubles the risk of obesity (44).

### Perceived Status of Overweight and Obesity

Overweight and obesity is a modifiable risk factor of vast diseases, especially NCDs with adverse consequences among the older generation. All FGDs in this research stated that the medical and social consequences of overweight and obesity are detrimental with chronic effects among the adult group and acute effects amid adolescents. As reported in many articles and research, NCDs with cardiorespiratory and orthopedic (45) disabilities are disheartening and common among adolescents and most recently adults (46). Thus, a common understanding of the perceived status of overweight and obesity is noted.

However, the actual question now is whether overweight and obesity is perceived as a modifiable risk factor to vast diseases? This study outlines that 65.6% of participants are overweight with 25% being obese. There is a perception-happening mismatch that frustrates the statement in Health Belief Model stating that if an individual perceives a problem or condition as severe, the normal response would be counteracting the condition (47). This study further reports that students who are overweight or obese do not seek immediate medical attention, and in most experiences, health clinic visits are done only when in critical stage. Similar studies highlighted the underestimation (48) and misperception (49) of weight as a common ambiguous reason. However, the strong cultural belief in Kiribati, as reported in this study, of being big is beautiful is a commonly recognized misperception of weight.

The evidence of this research suggests that consequences of overweight and obesity are perceived well among Kiribati population but not so much stabilized in the early stage. This calls for the development of strategic measures that are feasible in schools for students to practice and implement.

### Perceived Action Benefits

Reduced stress and anxiety, enhanced health, and improved self-confidence are the moral behind the immediate action benefits of overweight and obesity. These perceived benefits of actions as voiced and discussed by FGDs are an encouragement and motivation to those affected by weight control. As Phelan et al. mentioned in their study, the perceived benefits of weight control are an important predictor of intentional weight loss (50). Moreover, if the benefit of the preventative strategy is to improve or progress in a positive manner, the rate of participation may increase. As mentioned in the study by Korn et al., the adolescent's dietary behavior changes for better seeing the benefit it poses on physical appearance, health, and self-confidence (51). The results of this study contribute to the awareness of those with overweight and obese and volunteering to participate in available programs.

### Perceived Barriers to Practices

Overweight and obesity is a complex medical condition with superfluous consequences. However, overcoming these consequences initiates public comments that result in decreased self-esteem and psychological stress to victimized individuals. All groups in the FGD reported several attempts to exercise but are held back by shame and public comments. This shows that misconception of overweight and obesity exists among society; in fact, it exists in homes, schools, communities, and even in healthcare settings (52). To decrease weight stigma, everyone needs to be informed of the negative impact it has on victims of overweight and obesity as mentioned in the 2015 Milan Declaration to Action on Obesity (53).

### Proposed Strategies for the Prevention of Overweight and Obesity

Home improvement, school support, and national sustenance were highlighted strategies for the prevention of overweight and obesity. According to the study by Smith et al., 22 articles exist relating to home improvement for the prevention of obesity (54), while Showell et al. emphasized home-based overweight prevention as being most effective (55). Home improvement in terms of family support, food preparation techniques, having home gardens, and family discussion and meals were mentioned. Berge and Everts proved that any form of family-based intervention in obesity prevention was effective in 70% of the literature reviewed (56). Zhang et al. looked at food policy and its approach to obesity prevention. Results gained insight into the positive effect of implementing food policies (57).

Another finding is the school support and its effect on the prevention of overweight and obesity. Nihiser et al. highlighted similar school aspects, such as setting, policy and curriculum, and food and nutrition in overweight prevention (58). Kahan and McKenzie supported physical education and activity as a part of the school curriculum (59). While Li et al. studied the effect of school-based intervention, results concluded that it is effective in decreasing excess body fat (60).

With national sustenance, the issues of budget, public awareness, and stakeholder's involvement were reported and proposed. Yang and Hall mentioned that overweight and obesity was a financial burden to the nation (61). Agrawal et al. commented on awareness strategy among Indian women in relation to overweight prevention. Results highlighted the implementation of policies as being effective in preventative measures (62). As Ganter et al. stated, community stakeholders request preventative programs to be feasible when addressing obesity (63).

Perspectives of SCMs are broad, and this study has highlighted a few main findings that are open for future policy development and research.

### Strengths

This is the first study to investigate this rapidly growing health issue in Kiribati, and it is important to gain data on options/feasibility of taking beneficial action at the school level. This study also used a qualitative approach to gain bottom-up perceptions/knowledge of local people rather than a more typical top-down researcher-determined survey questionnaire approach. It is particularly important for tapping into local cultural beliefs and practices that might facilitate or hinder action, e.g., “Big is beautiful” the local cultural attitude.

### Limitations

Due to the cultural characteristics of people in Kiribati, some participants may not have felt comfortable in sharing their accounts in English, and thus, Kiribati language was used as the primary communication. Moreover, SCMs were held after hours for FGDs which may have limited their time to engage and express their opinions.

## Conclusion

The factors contributing to overweight and obesity among high school students are vast, and this research has identified the main message constituted in bridging the knowledge-behavior gap among high school students with constructive influence from SCMs. Furthermore, improving public awareness will improve on the six themes founded from this study.

The recommendation is to recognize and comprehend the significance of the school-based approach in preventing overweight and obesity among adolescents where student's time, availability of expertise, and influence of peers all exist will bring in the concept of a multifactorial approach to a multifaceted cause. Interventions in schools will need to look at structural variables needing school setting regulation or policy that investigates the availability of healthy foods or exercise equipment, in addition to the knowledge and behavior of school children, and to encourage physical exercise and healthy diet in schools nationally to promote good health, behaviors, and lifestyles.

## Data Availability Statement

The raw data supporting the conclusions of this article will be made available by the authors, without undue reservation.

## Ethics Statement

The studies involving human participants were reviewed and approved by the College Health Research Ethics Committee (CHREC) in Fiji National University (FNU). The patients/participants provided their written informed consent to participate in this study.

## Author Contributions

TT developed proposal, collected data, and analyzed data. All authors have contributed to the concept, design, writing, and revising of the manuscript and have also approved for the submission.

## Conflict of Interest

The authors declare that the research was conducted in the absence of any commercial or financial relationships that could be construed as a potential conflict of interest.

## Publisher's Note

All claims expressed in this article are solely those of the authors and do not necessarily represent those of their affiliated organizations, or those of the publisher, the editors and the reviewers. Any product that may be evaluated in this article, or claim that may be made by its manufacturer, is not guaranteed or endorsed by the publisher.

## References

[1] Karnik S Kanekar A. Childhood obesity: a global public health crisis. Int J Prev Med. (2012) 3:1–7. 22506094 PMC3278864

[2] Vos MB Welsh J. Childhood obesity: update on predisposing factors and prevention strategies. Curr Gastroenterol Rep. (2010) 12:280–7. 10.1007/s11894-010-0116-1 20563673 PMC3056648

[3] Yatsuya H Li Y Hilawe EH Ota A Wang C Chiang C. Global trend in overweight and obesity and its association with cardiovascular disease incidence. Circ J. (2014) 78:2807–18. 10.1253/circj.CJ-14-0850 25391910

[4] Al-Hazzaa HM Abahussain NA Al-Sobayel HI Qahwaji DM Musaiger AO. Physical activity, sedentary behaviors, and dietary habits among Saudi adolescents relative to age, gender, and region. Int J Behav Nutr Phys Act. (2011) 8:140. 10.1186/1479-5868-8-140 22188825 PMC3339333

[5] Rathnayake KM Roopasingam T Wickramasighe VP. Nutritional and behavioral determinants of adolescent obesity: a case-control study in Sri Lanka. BMC Public Health. (2014) 14:1291. 10.1186/1471-2458-14-1291 25519979 PMC4302095

[6] Rodrigues PRM Luiz RR Monteiro LS Ferreira MG Gonçalves-Silva RMV Pereira RA. Adolescents' unhealthy eating habits are associated with meal skipping. Nutrition. (2017) 42:114–20.e1. 10.1016/j.nut.2017.03.011 28596058

[7] Djalalinia S Qorbani M Peykari N Kelishadi R. Health impacts of obesity. Pak J Med Sci Q. (2015) 31:239–42. 10.12669/pjms.311.7033 25878654 PMC4386197

[8] Xu Q Zhou M Jin D Zeng X Qi J Yin L. Projection of premature mortality from noncommunicable diseases for 2025: a model-based study from Hunan Province, China, 1990-2016. Peer J. (2020) 8:e10298. 10.7717/peerj.10298 33194444 PMC7646306

[9] Khang Y-H. Burden of noncommunicable diseases and national strategies to control them in Korea. J Prev Med Public Health. (2013) 46:155–64. 10.3961/jpmph.2013.46.4.155 23946873 PMC3740220

[10] Chan RSM Woo J. Prevention of overweight and obesity: how effective is the current public health approach. Int J Environ Res Public Health. (2010) 7:765–83. 10.3390/ijerph7030765 20617002 PMC2872299

[11] Panuganti KK Nguyen M Kshirsagar RK. Obesity. In: StatPearls. Treasure Island, FL: StatPearls Publishing (2020).

[12] Pengpid S Peltzer K. Overweight and obesity and associated factors among school-aged adolescents in six Pacific Island countries in Oceania. Int J Environ Res Public Health. (2015) 12:14505–18. 10.3390/ijerph121114505 26580638 PMC4661663

[13] Asia E Edition PR. The Global Burden of Disease: Generating Evidence, Guiding Policy Available online at: https://documents1.worldbank.org/curated/fr/758591468027855762/pdf/808500PUB0L2 590Box0379820B00PUBLIC0.pdf (accessed September 21, 2020).

[14] World Health Organization. Global Status Report on NCDs. (2015) Available online at: http://www.who.int/chp/ncd_global_status_report/en/ (accessed Jul 21, 2021).

[15] Pengpid S Peltzer K. Childhood overweight and social correlates among school-going adolescents in Dominica and Jamaica. Afr J Phys Act Health Sci. (2014) 20:636–45.

[16] Samaranayaka S Perera A Warnasuriya N. Food habits among adolescents in colombo, Sri Lanka. World Fam Med J Middle East J Fam Med. (2013) 11:26–34. 10.5742/MEJFM.2013.116277

[17] Mahmood S Perveen T Dino A Ibrahim F Mehraj J. Effectiveness of school-based intervention programs in reducing prevalence of overweight. Indian J Community Med. (2014) 39:87–93. 10.4103/0970-0218.132724 24963224 PMC4067935

[18] Robinson TN. Reducing children's television viewing to prevent obesity: a randomized controlled trial. JAMA. (1999) 282:1561–7. 10.1001/jama.282.16.1561 10546696

[19] Harrell JS McMurray RG Bangdiwala SI Frauman AC Gansky SA Bradley CB. Effects of a school-based intervention to reduce cardiovascular disease risk factors in elementary-school children: the Cardiovascular Health in Children (CHIC) study. J Pediatr. (1996) 128:797–805. 10.1016/S0022-3476(96)70332-3 8648539

[20] Doak CM Visscher TLS Renders CM Seidell JC. The prevention of overweight and obesity in children and adolescents: a review of interventions and programmes. Obes Rev. (2006) 7:111–36. 10.1111/j.1467-789X.2006.00234.x 16436107

[21] Jourdan D Samdal O Diagne F Carvalho GS. The future of health promotion in schools goes through the strengthening of teacher training at a global level. Promot Educ. (2008) 15:36–8. 10.1177/1025382308095657 18784053

[22] Bucher Della Torre S Akré C Suris J-C. Obesity prevention opinions of school stakeholders: a qualitative study. J Sch Health. (2010) 80:233–9. 10.1111/j.1746-1561.2010.00495.x 20529196

[23] Herbert D. School Choice in the Local Environment: headteachers as gatekeepers on an uneven playing field. Sch Lead. Manag. (2000) 20:79–97. 10.1080/13632430068897

[24] Howard-Drake EJ Halliday V. Exploring primary school headteachers' perspectives on the barriers and facilitators of preventing childhood obesity. J Public Health. (2016) 38:44–52. 10.1093/pubmed/fdv021 25750005

[25] Ganter C Aftosmes-Tobio A Chuang E Blaine RE Land T Davison KK. Community stakeholders' perceptions of major factors influencing childhood obesity, the feasibility of programs addressing childhood obesity, and persisting gaps. J Community Health. (2016) 41:305–14. 10.1007/s10900-015-0097-y 26433725 PMC6555410

[26] Site designed, built by, Hydrant. Kiribati : History. Thecommonwealth.org (2019). Available online at: (http://www.hydrant.co.uk; https://thecommonwealth.org/our-member-countries/kiribati/history (accessed Jul 7, 2021).

[27] The 2018 household listing for Kiribati. Spc.int. Available online at: https://sdd.spc.int/collection/2018-household-listing-kiribati (accessed Jul 7, 2021).

[28] Carter KL Baiteke T Teea T Tabunga T Itienang M Rao C. Mortality and life expectancy in Kiribati based on analysis of reported deaths. Popul Health Metr. (2016) 14:3. 10.1186/s12963-016-0072-6 26933387 PMC4772294

[29] Kiribati Demographics,. Worldometers.info. (2020). Available online at: https://www.worldometers.info/demographics/kiribati-demographics/ (accessed Jul 7, 2021).

[30] LK. Map of Kiribati. Besthotelshome.com (2020). Available online at: https://besthotelshome.com/map-of-kiribati/ (accessed Jul 7, 2021).

[31] Bree RT Gallagher G. Using Microsoft Excel to code and thematically analyse qualitative data: a simple, cost-effective approach. AISHE J. (2016) 8:2811–20.

[32] Reed MC. Book Reviews : Fourth Generation Evaluation by Egon Guba and Yvonne Lincoln. Newburg Park, CA: Sage (1989). p. 294.

[33] Levy TS. Overweight and obesity: are they an irremediable situation? Bol *Méd Hosp Infant Méx*. (2016) 73:65–6. 10.1016/S2444-3409(16)30001-2 29421196

[34] Robinson TN Banda JA Hale L Lu AS Fleming-Milici F Calvert SL. Screen media exposure and obesity in children and adolescents. Pediatrics. (2017) 140(Suppl. 2):S97–101. 10.1542/peds.2016-1758K 29093041 PMC5769928

[35] Hruby A Manson JE Qi L Malik VS Rimm EB Sun Q. Determinants and consequences of obesity. Am J Public Health. (2016) 106:1656–62. 10.2105/AJPH.2016.303326 27459460 PMC4981805

[36] Spahlholz J Baer N König H-H Riedel-Heller SG Luck-Sikorski C. Obesity, and discrimination - a systematic review and meta-analysis of observational studies: obesity and discrimination. Obes Rev. (2016) 17:43–55. 10.1111/obr.12343 26596238

[37] Jung FU Luck-Sikorski C. Overweight and lonely? A representative study on loneliness in obese people and its determinants. Obes Facts. (2019) 12:440–7. 10.1159/000500095 31315116 PMC6758716

[38] Malcolm M Frost H Cowie J. Loneliness, and social isolation causal association with health-related lifestyle risk in older adults: a systematic review and meta-analysis protocol. Syst Rev. (2019) 8:48. 10.1186/s13643-019-0968-x 30732659 PMC6366024

[39] Hosseini SA Padhy RK. Body image distortion. In: StatPearls. Treasure Island, FL: StatPearls Publishing (2021). 31536191

[40] Voelker DK Reel JJ Greenleaf C. Weight status and body image perceptions in adolescents: current perspectives. Adolesc Health Med Ther. (2015) 6:149–58. 10.2147/AHMT.S68344 26347007 PMC4554432

[41] Zainal Abidin N Mamat M Dangerfield B Zulkepli JH Baten MA Wibowo A. Combating obesity through healthy eating behavior: a call for system dynamics optimization. PLoS ONE. (2014) 9:e114135. 10.1371/journal.pone.0114135 25502170 PMC4266604

[42] Borraccino A Lemma P Berchialla P Cappello N Inchley J Dalmasso P. Unhealthy food consumption in adolescence: role of sedentary behaviours and modifiers in 11-, 13- and 15-year-old Italians. Eur J Public Health. (2016) 26:650–6. 10.1093/eurpub/ckw056 27085192 PMC5885947

[43] Silva DFO Sena-Evangelista KCM Lyra CO Pedrosa LFC Arrais RF Lima SCVC. Motivations for weight loss in adolescents with overweight and obesity: a systematic review. BMC Pediatr. (2018) 18:364. 10.1186/s12887-018-1333-2 30463551 PMC6247735

[44] Mühlig Y Wabitsch M Moss A Hebebrand J. Weight loss in children and adolescents. Dtsch Arztebl Int. (2014) 111:818–24. 10.3238/arztebl.2014.0818 25512008 PMC4269075

[45] Birchall H. Handbook of obesity treatment: book reviews. Eur Eat Disord Rev. (2002) 10:376–376. 10.1002/erv.48212527156

[46] Niehoff V. Childhood obesity: a call to action. Bariatr Nurs Surg Patient Care. (2009) 4:17–23. 10.1089/bar.2009.9996

[47] American Academy of Pediatrics. About Childhood Obesity. (2014). Available online at: http://www.aap.org/obesity/about.html (accessed December 20, 2020).

[48] Rosenstock IM. Why people use health services: why people use health services. Milbank Q. (2005) 83:1–32. 10.1111/j.1468-0009.2005.00425.x5967464

[49] Visscher TLS Viet AL Kroesbergen IHT Seidell JC. Underreporting of BMI in adults and its effect on obesity prevalence estimations in the period 1998 to 2001. Obesity. (2006) 14:2054–63. 10.1038/oby.2006.240 17135623

[50] Mogre V Nsoh JA Wanaba P Apala P. Demographic factors, weight management behaviours, receipt of healthcare professional's counselling and having knowledge in basic anthropometric measurements associated with underassessment of weight status in overweight and obese type 2 diabetes patients. Obes Res Clin Pract. (2016) 10:381–9. 10.1016/j.orcp.2015.08.018 26385600

[51] Phelan S Wing RR Loria CM Kim Y Lewis CE. Prevalence and predictors of weight- loss maintenance in a biracial cohort: results from the coronary artery risk development in young adults' study. Am J Prev Med. (2010) 39:546–54. 10.1016/j.amepre.2010.08.008 21084075 PMC3308341

[52] Korn L Gonen E Shaked Y Golan M. Health perceptions, self and body image, physical activity and nutrition among undergraduate students in Israel. PLoS ONE. (2013) 8:e58543. 10.1371/journal.pone.0058543 23516503 PMC3597731

[53] Puhl RM Heuer CA. Obesity stigma: important considerations for public health. Am J Public Health. (2010) 100:1019–28. 10.2105/AJPH.2009.159491 20075322 PMC2866597

[54] European Association for the Study of Obesity. Milan Declaration: A Call to Action on Obesity. A Statement of the Members of the European Association for the Study of Obesity to EXPO (2015). Available online at: http://gucdv1wwi8pslzdfpv7t0dk6.wpengine.netdna-cdn.com/wp- (accessed January 21, 2021).

[55] Smith JD Fu E Kobayashi MA. Prevention and management of childhood obesity and its psychological and health comorbidities. Annu Rev Clin Psychol. (2020) 16:351–78. 10.1146/annurev-clinpsy-100219-060201 32097572 PMC7259820

[56] Showell NN Fawole O Segal J Wilson RF Cheskin LJ Bleich SN. A systematic review of home-based childhood obesity prevention studies. Pediatrics. (2013) 132: e193–200. 10.1542/peds.2013-0786 23753095 PMC3691540

[57] Berge JM Everts JC. Family-based interventions targeting childhood obesity: a meta- analysis. Child Obes. (2011) 7:110–21. 10.1089/chi.2011.07.02.1004.berge 26182126 PMC4504253

[58] Zhang Q Liu S Liu R Xue H Wang Y. Food policy approaches to obesity prevention: an international perspective. Curr Obes Rep. (2014) 3:171–82. 10.1007/s13679-014-0099-6 25705571 PMC4333683

[59] Nihiser A Merlo C Lee S. Preventing obesity through schools. J Law Med Ethics. (2013) 41:27–34. 10.1111/jlme.12106 24446995 PMC4605133

[60] Kahan D McKenzie TL. The potential and reality of physical education in controlling overweight and obesity. Am J Public Health. (2015) 105:653–9. 10.2105/AJPH.2014.302355 25713972 PMC4358179

[61] Li X-H Lin S Guo H Huang Y Wu L Zhang Z. Effectiveness of a school-based physical activity intervention on obesity in school children: a nonrandomized controlled trial. BMC Public Health. (2014) 14:1282. 10.1186/1471-2458-14-1282 25510313 PMC4320634

[62] Yang Z Hall AG. The financial burden of overweight and obesity among elderly Americans: the dynamics of weight, longevity, and health care cost: overweight and obesity among elderly Americans. Health Serv Res. (2008) 43:849–68. 10.1111/j.1475-6773.2007.00801.x 18454771 PMC2442233

[63] Agrawal S Agrawal P Gupta K Mishra V. Awareness on causes, consequences and preventive measures of obesity among urban married women in India. Int J Med Public Health. (2013) 3:293. 10.4103/2230-8598.123476 28856116 PMC5573173

